# Abuse of Purgative Medicines

**Published:** 1876-03

**Authors:** 


### ABUSE OF PURGATIVE MEDICINE.

Dr. Bodenhamer, in his valuable work on the Diseases of the Rectum, makes the following remarks in reference to an often and serious cause of such maladies:

"The abuse of purgative medicine is, at the present day, a frequent cause of anal and rectal diseases. The idea that it is either necessary to obviate constipation of the bowels, or on every slight indisposition to swallow some of the numerous and various purgative nostrums which literally fill the shelves of our drug-stores, is a popular error, and a source of incalculable mischief. It has laid the foundation of some of the most serious diseases of the lower viscera. The habitual use of such medicines to obviate constipation, I repeat, is the cause of more diseases of the anal region than any other one cause that has come under my observation. I have ascertained to a certainty, that in a very large number of the cases I treat for such diseases, the cause can distinctly be traced to this origin.

"The continued exhibition of such medicines, inflicts much reckless and unnecessary injury, by the undue and pernicious excitement of the whole intestinal canal which is thus induced, and all for the purpose merely of emptying the rectum and forcing the dilatation of the anus, which, after all, are accomplished at the expense of the intestinal fluids and the softening and the washing out of the excrementitious matters. In all such cases, a proper attention to diet, to exercise, and to the occasional use of an enema of cold water, or flaxseed tea, would obviate the difficulty without inflicting any injury whatever. It is, however, by no means easy to convince some people that such medicine can not safely be made use of as a substitute for moderation in diet, for pure air, and the proper exercise of the whole muscular system—in short, for all the *natural* measures which long experience has shown to be necessary for the preservation of health. They sometimes experience much relief, much comfort from the operation of the medicine, especially after having suffered for several days from constipation; but this immunity from discomfort is but transitory and deceptive. The same difficulty soon returns with increased force, and the same remedy must again be resorted to, and in order to produce the same effect, must either be increased in quantity or in strength at each repetition. It is the pleasant feeling, or the exhilaration which is often experienced by this class of invalids immediately after the free operation of purgative medicine, which induces them to repeat the same on each and every recurrence of the constipation or indisposition."

"A few years ago," says Quain, in his work on Diseases of the Rectum, "a case came to my knowledge which will serve to illustrate the baneful influence of the habit of using purgative medicine. The commander of a merchant-vessel, a person of robust frame and much ability in his profession, began to take Morrison's pills to relieve constipation of the bowels at sea. Continuing the use of the medicine, he became in time reduced to extreme debility from constant purging. At length the appetite grew by what it fed on, to such an extent, that when confined to his bed from mere weakness, and unable to swallow the pills whole, the unhappy man had them bruised in a mortar, and took them with a spoon. He died of the drug."

Dr. J. B. Flint of Louisville, Ky., says that "Nothing has been more remarkable in my surgical experience in the West, than the disproportioned frequency of diseases of the rectum and adjacent textures—fistula, piles, prolapsus, etc.—and I advert to the fact chiefly for the purpose of adding a cautioning remark respecting the causes of it. Doubtless it is partly to be referred to the chafing and contusions incident to horseback-riding, which is a much more common mode of traveling here than at the East; but it is mainly attributable to the habit of indiscriminate and excessive purgation, so prevalent both as a remedial and prophylactic measure.

"A large portion of the practitioners of the valley of the Mississippi have been educated under a system of medicine whose theory regards portal congestion and hepatic derangement, as the essential elements of all diseases, and whose practice consists, almost exclusively, in the exhibition of drastic purgatives. It is natural that the people should imitate the therapeutics of their medical advisers, when so simple and easily applied; accordingly they are as much in the habit of drenching themselves and teasing the alimentary canal, on every occasion of illness, with some concentrated purgative in the form of pills.

"Under one of the most constant laws of irritation in mucous canals, the terminating portions of the apparatus of defecation are thus perpetually suffering under propagated, as well as direct stimulation, and reäcts in the various forms of disease under notice. Besides these direct mischiefs and others, involving the health in other ways, occasioned by the pernicious doctrines referred to—which are indeed in themselves essentially empirical — they encourage the grossest species of quackery by promoting the consumption of vast quantities of patent pills and other purgative nostrums.

"Several years since, a gentleman from one of the Southern States consulted me for a *fistula in ano* which caused him much suffering. He stated that for a year or two previous to the formation of the fistula, he could never have an evacuation from his bowels without swallowing great quantities of Brandreth's pills; that he frequently took as many as thirty and forty at one dose; 'and,' said he, 'when they did commence to *work*, they operated like a *saw-mill*.' This is but one example out of hundreds that I might give to demonstrate the injurious effects of this pernicious practice.

"The motto of most all the quacks of the present day for the *cure of all diseases*, is PHYSIC! PHYSIC!! PHYSIC!!! Purgative medicines are good in their proper place, but to purge for every thing is absolutely absurd; therefore all such purgative nostrums, in the form either of *pills*, *bitters*, *mineral waters*, or any thing else, should be eschewed as the cholera.

"Nearly all the nostrums or patent-right medicines which now fill the shelves of our drug-shops are founded by their authors upon the principle that *disease is a unit;* that there is but *one general cause* of disease, and but *one general remedy*, and that is always certain to be their own infallible and peculiar one; hence their *panaceas*, *blood-purifiers*, *elixirs of life*, etc., in the form of *pills*, *bitters*, *sarsaparilla syrups*, etc., etc. It is surprising how popular this theory is among the masses, and even among physicians. Those minds which are but superficially informed and unaccustomed to the slow and gradual progress of inductive science are readily seduced and captivated by the reasoning of those who advocate this pernicious system.

The best remedy we have ever employed for constipation and piles is "NELATON'S SUPPOSITORY."

